# Dynamic Opportunities for Medical Students to Assume the Roles of “Medical Teacher”

**DOI:** 10.1007/s40670-023-01969-8

**Published:** 2024-01-25

**Authors:** Chitra Kumar, Avery Miller, Aaron M. Marshall, Andrew R. Thompson, D. J. Lowrie, Danielle E. Weber, Matt Kelleher, Laura Malosh, Carrie Martin, Heather R. Christensen

**Affiliations:** 1 The University of Cincinnati College of Medicine, Cincinnati, OH USA; 2 Department of Medical Education, 231 Albert Sabin Way, Cincinnati, OH 45267 USA; 3 Department of Pediatrics, 231 Albert Sabin Way, Cincinnati, OH 45267 USA; 4 Department of Internal Medicine, 231 Albert Sabin Way, Cincinnati, OH 45267 USA

**Keywords:** Teaching, Elective, Student-teachers, Undergraduate medical education

## Abstract

The traditional undergraduate medical education curriculum focuses on bolstering knowledge for practice and building clinical skills. However, as future clinicians, medical students will be tasked with teaching throughout their careers, first as residents and then as attendings. Here, we describe teaching opportunities for students that foster their development as future teachers and potential clinician educators. These offerings are diverse in their focus and duration and are offered across various levels of the curriculum — including course-based learning, longitudinal electives, and extra-curricular opportunities for medical students who have a passion for teaching.

## Introduction 

Teaching is a fundamental component of the medical profession, requiring students to serve as educators throughout their training. Fourth-year students (M4s) often assist third-year students (M3s) throughout the core clerkships. Because time constraints and increasing economic pressures on the healthcare system decrease the time attendings can spend with medical students, residents play an increasingly significant role in medical education [1–3]. Senior residents supervise and help junior residents hone their skills, preparing them to become senior residents. When trainees become attendings, teaching is already an integral part of their physician role. Considering the significant role teaching plays in the development of future physicians, early training in educational principles during medical school can improve effective communication from teachers while increasing learning in their students [4]. In addition, teaching opportunities during pre-clerkship years allow students to practice teaching skills while receiving constructive, standardized feedback in a controlled environment, which can be drawn upon to improve their skills before they become resident teachers [4].

The need for formal instruction in educational practice across the continuum of medical education (MedEd) is recognized by both teachers and learners [5]. A study by Huynh et al*.* showed a student’s perception of a specialty, and the quality of their learning experience is strongly affected by a resident’s ability to teach [6]. Residents have reported they would prefer more opportunities to learn how to teach [7]. The ability to teach effectively is a critical skill for clinicians — one that needs to be taught with intention and should be fostered in medical school. Indeed, effective teaching is complex, requiring skills across many arenas. Harden and Crosby offer a framework regarding the multiple roles of a medical teacher [8], which is helpful when considering how to motivate and train the next generation of physician educators. They describe twelve roles of the medical teacher, grouped into six areas (information provider, role model, facilitator, assessor, planner, and resource developer), which we refer to in this article as we describe the impact of each student-as-teacher offering.

Authors such as Cohen et al*.* have published systematic reviews on curricula where students are also teachers [9]. Unsurprisingly, multiple medical institutions already incorporate peer teaching through journal clubs, tutoring, anatomy lab supplementation, biostatistics modules, and point-of -care ultrasound [10–15]. In some instances, institutions (including UCCoM) create teaching opportunities out of necessity (i.e., when recruiting an adequate number of faculty teachers is prohibitive); even in such cases, however, students-as-teachers are being provided an opportunity to step into an educator role. Herein, we detail comprehensive initiatives at the University of Cincinnati College of Medicine (UCCoM) to train medical students to become effective teachers as they prepare for a career in medicine.

## Overview of the University of Cincinnati Medical School Curriculum

The UCCoM curriculum is divided into pre-clerkship years (first and second year, M1 and M2, respectively), a clerkship year (M3), and a post-clerkship year (M4), with approximately 185 students per cohort. Using an integrated curricular approach, including laboratory, small group discussions, team-based learning, and lectures, the pre-clerkship years provide students with scientific and humanistic principles of medicine. Clinical exposure begins in M1: standardized patient encounters in a clinical skills laboratory (team-based) and an 18-month longitudinal clinical experience at an outpatient primary care site. During M3, students rotate through seven required core clerkships and explore career options through subspecialty electives. M4 includes two required “acting internships” (AIs) and over 100 elective offerings for continued specialty exploration.

## Teaching Opportunities, from Course to Curriculum

**Fig. 1:**
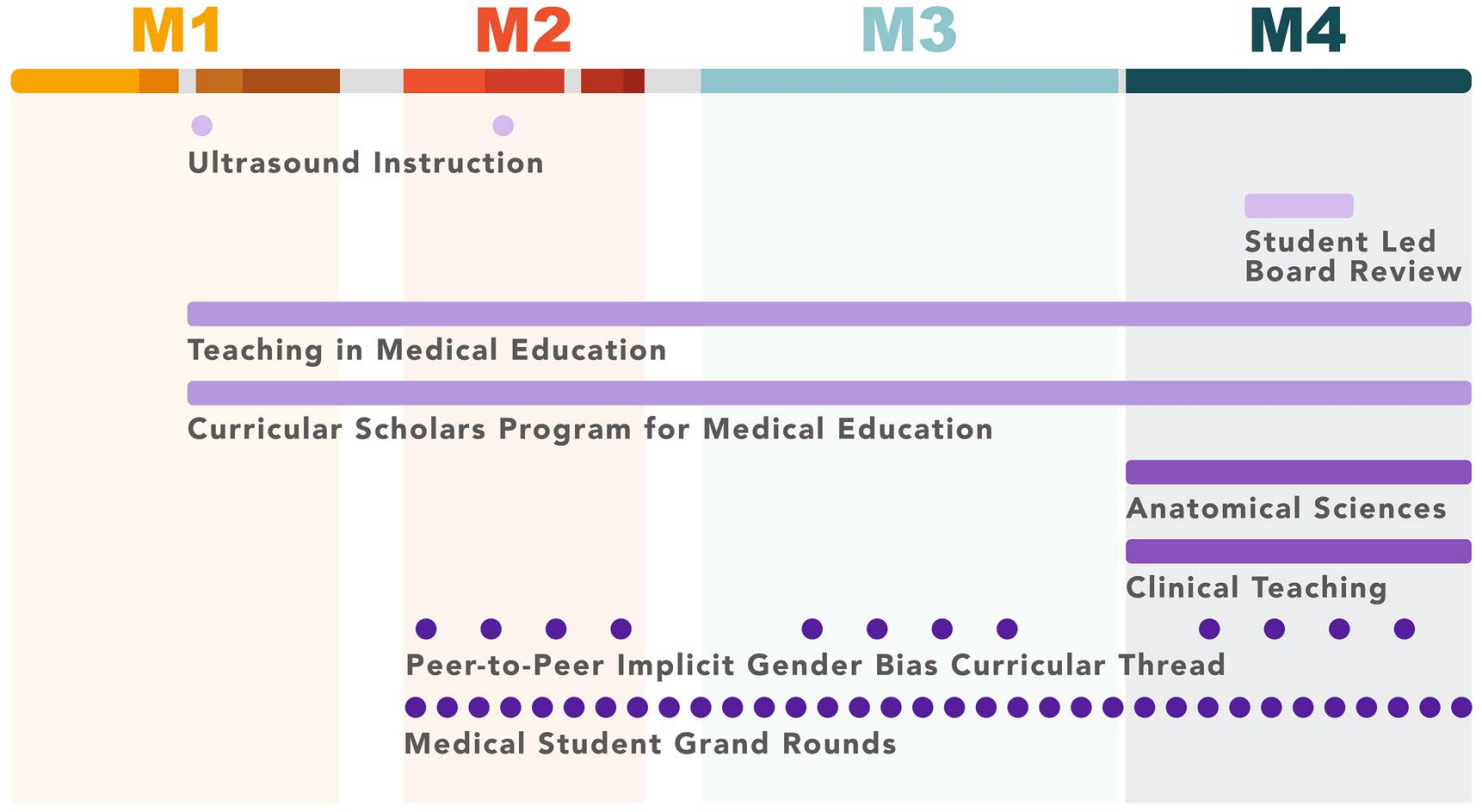
Timeline highlighting teaching opportunities for students in the UCCoM undergraduate medical education curriculum. Course years (M1 through M4) are indicated across the top and shaded for the length of the different foundational courses in that all students take simultaneously during M1/M2 years. Clerkship experiences (M3) and post-clerkship learning (M4) are different for each learner. During the curriculum, individual opportunities for students to become teachers are represented as dot longitudinal experiences are represented as lines (from when the student first joins the teaching experience until the experience concludes)

For students interested in developing skills as an educator, UCCoM offers several opportunities throughout the undergraduate MedEd (UME) curriculum which allow students to study and practice teaching (Fig. 1). These include teaching in anatomy laboratories, longitudinal electives, and electives confined to post-clerkship year M4, as well as formal tutoring programs.

**Fig. 2:**
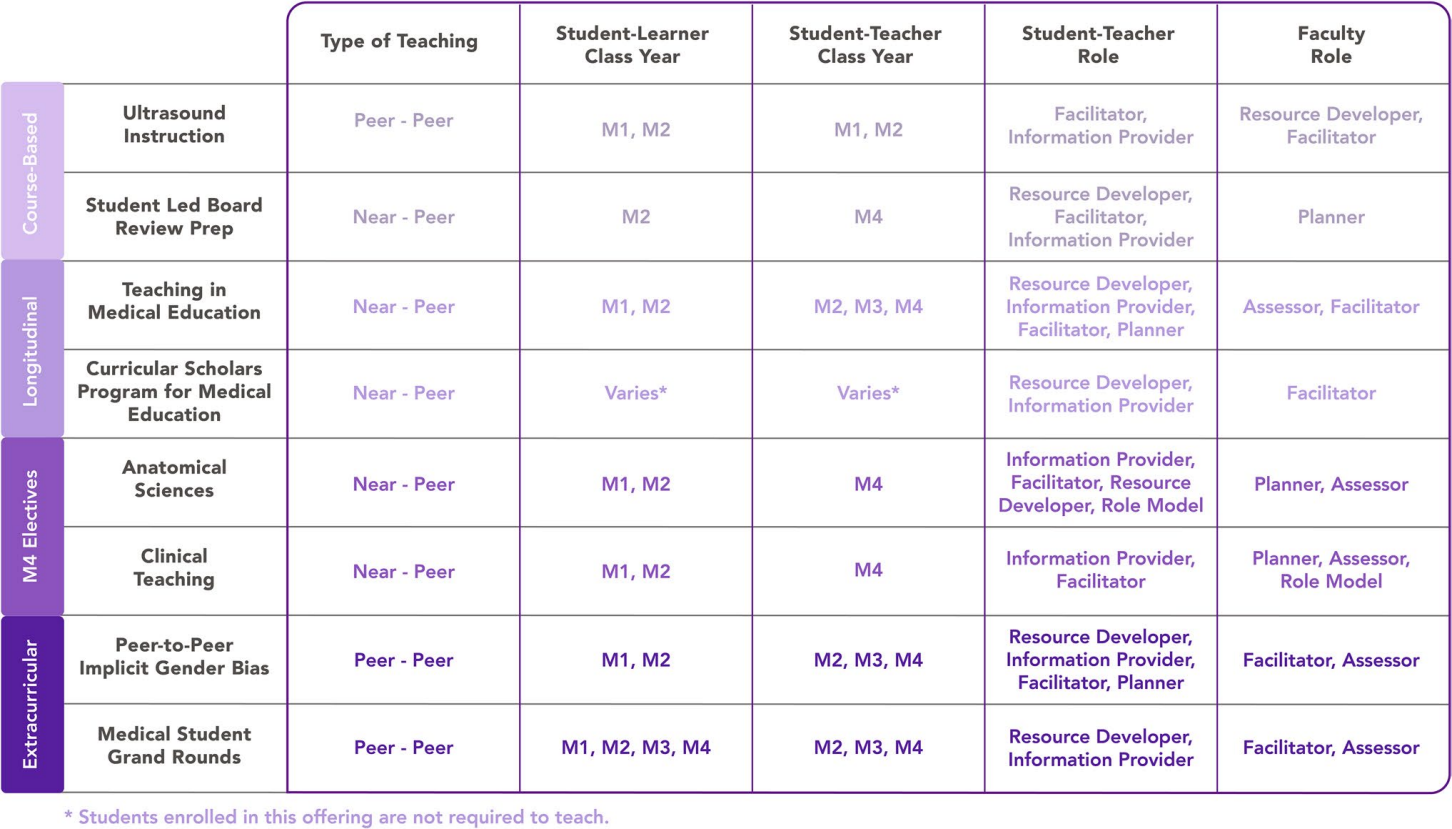
Multiple teaching experiences provide opportunities to practice various roles of a medical teacher. The UCCoM teaching offerings (listed on the left) are detailed by the type of teaching taking place, the class year for the student–learner and student–teacher, and the teacher role for the student–teacher role and faculty [8]

The purpose of this manuscript is to provide descriptions of these teaching opportunities, organized by the point and duration at which they are integrated into our MedEd curriculum. For each, we highlight the student–teacher–learner relationship (Fig. 2) and, when applicable, discuss the impact of the experience and challenges encountered in the development or implementation of said offerings. Utilizing the aforementioned framework [8], we highlight the educator roles students are able to practice (i.e., developing resources, planning/facilitating, and serving as role models for their peers) (Fig. 2). Notably, one educator role students do not formally encounter through our teaching opportunities is “assessor.” Student-teachers may informally practice assessing their learners using practice questions within a session, but the faculty do not robustly train students in item writing. This is, in part, due to the intricacies involved with effective assessment of learning. In fact, the training and practice for the role of assessor were described by Harden and Crosby to be perhaps the “greatest challenge” to MedEd, persisting since they first described these roles in 2000 [8]. Thus, it is appropriate that early trainees do not assume that role.

We aim to provide readers examples of how teacher training opportunities can be implemented in a UME curriculum, spanning the spectrum from individual teaching events to curriculum scholar programs while addressing a gap (elevating students to the role of teacher) that currently exists in MedEd [5]. Whether a curriculum leader, educator faculty, or medical student, we hope this inspires health professions institutions to adopt similar opportunities.

## Course-Level Peer Teaching in the Pre-Clerkship Curriculum

Providing formal opportunities for students to learn and hone teaching skills at the course-level during a UME pre-clerkship phase is challenging, particularly given the volume and density of material students must navigate as learners. One solution is to create micro-opportunities for teaching within a course, utilizing a peer teaching (PT) model. Fundamentally, PT involves students assuming the role of teachers to instruct peers at the same general academic level. When upper-level students serve as teachers, that instruction is more specifically called “near-peer teaching” (NPT).

In UME, one of the most common applications of a PT/NPT model is within laboratory-based gross anatomy instruction; student teams alternate dissection responsibilities, and then peer teach the anatomy to those who did not dissect. This PT approach has largely positive outcomes [16–19], without adding significant burden to the student. Given the broadly utilized and well-published nature of PT in dissection, our manuscript instead focuses on unique opportunities to utilize PT within pre-clerkship learning. Broadly, the activities described below provide students an opportunity (albeit without formal training) to assume the roles of information provider and/or facilitator (Fig. 2), utilizing resources designed by faculty. Here, assessment of learning occurs outside of the actual teaching event.

### Ultrasound Education

Recent studies show increased incorporation of ultrasound (US) education into pre-clerkship curricula [20, 21]. For these learning events, which are most effective with small learner-to-teacher ratios, hands-on instruction is critical [15]. As such, a major barrier educators face when designing US learning activities is recruiting an adequate number of teaching faculty trained in US [22, 23]. One commonly practiced, effective solution to this problem is to utilize residents or M3/M4 students as NPTs to train pre-clerkship medical students in US [15, 24–27]. Indeed, UCCoM residents in physical medicine and rehabilitation (PMR) are recruited as NPT in an US session that focuses on the shoulder examination. However, even with residents and UME faculty, we could not meet the desired student-to-teacher ratio.

To combat this teacher deficiency and provide a novel teaching experience, we embraced a hybrid approach of both NPT and PT. M1s enrolled in the musculoskeletal course were provided the opportunity to volunteer to be instructors during US learning sessions (for M1s). The ultimate goal was a student-to-instructor ratio below 5:1, which has been shown to provide better learning outcomes [15].

M1 student-teachers attended a 1-h training session, facilitated by a PMR faculty experienced in musculoskeletal US. During this training, students observed and practiced the shoulder US examination, took notes, and asked questions. The initial hands-on US laboratory session for the entire M1 cohort was deliberately constructed with fewer learning groups and extended time; this allowed teaching faculty and PMR residents to observe and provide verbal and skill-based feedback to M1 PTs. After one round of teaching, M1 student-teachers expressed enough confidence to teach independently, alleviating faculty and residents, who could then observe (vs. directly teach) or excuse themselves from the session.

Our approach solved a common problem faced when implementing US education (limited teacher resources). The training to prepare M1s for this focused content was minimal, placing low demand on the faculty and a low cognitive load on student-teachers. Thus, this was an ideal low-stakes opportunity for teaching practice. Formal evaluation and assessment of student-teaching were not required, as student-teachers received adequate feedback in the moment, making necessary adjustments. This experience provided medical students a unique opportunity to gain valuable experience as learning facilitators [8], gaining a small introduction to MedEd practice.

### Student-Led Board Review Preparation Course

UCCoM’s Student-Led Board Review Preparation (SLBRP) provides an in-depth approach to assessment questions students will encounter on the US Medical License Exam STEP 1 board examination, which takes place at the culmination of our M2 curriculum. This optional program for M2 learners provides structure and strategy for completing board-style practice questions. Designed to strengthen students’ critical reasoning skills and systematic approach to test questions, one primary goal of the program is to review foundational content from M1/2 years. Secondarily, SLBRP aims to decrease anxiety associated with standardized exams.

To accomplish this, M4 student-teachers are recruited to teach small groups (six M2 student-learners) in an NPT learning environment (9 consecutive weeks, 2 h/week). M4 facilitators are trained by a learning specialist on evidence-based approaches to answering board-style questions. Group facilitators are given access to a bank of board practice questions, which they provide to and review with M2 learners. These questions include high-yield topics that reflect the breadth and depth of pre-clerkship content.

This NPT opportunity provides upper-level students the chance to facilitate learning and provide information for foundational concepts (Fig. 2) [8]. The “train-the-trainer” model by which one faculty prepares all student-teachers in the program is beneficial for a resource-limited institution. Some challenges do exist; namely, the coordination of student schedules (learners and teachers) is difficult. Despite scheduling each learning session at a consistent time, student-learner attendance varies, impacting the M4 student-teacher experience, as group composition influences both teaching and learning. Teaching consistency and effectiveness can also be a challenge, as some M4 facilitators may prioritize residency interview preparation.

## Dedicated Teaching Electives in the Post-Clerkship Curriculum

Post-clerkship medical student training provides opportunity to learn about and practice teaching after students have gained clinical experience prior to when formal teaching begins in residency). UCCoM offers teaching electives to M4s, focusing on pre-clinical or clinical content. One advantage of these teaching opportunities is the comfort of both the student–teacher (supervised by familiar faculty) and the student-learners (who have shared experiences with M4 NPTs). M4 students add value in that they can speak to the broad experience of medical students and share practical application of learned content that they themselves have only just encountered during clinical rotations. In this way, M4s can empathize with circumstances that faculty are farther removed from (i.e., giving oral clerkship presentations). M4s are also uniquely poised to answer questions and describe resources that resonate with student learners (i.e., an M4 just finishing a surgery rotation might better underscore clinical relevance of anatomical structures, or they might highlight the importance of laboratory tests learned in lecture after utilizing them directly in patient care on an internal medicine rotation). The learner–teacher relationship is bolstered by the fact that the M4s emphasizing these points are also still learners.

Both UCCoM electives described below provide M4 students opportunities to teach and were built upon an understanding that teaching is an important skill for residency. As mentioned above, utilizing M4 students-as-teachers is a benefit when faculty time and resources are sparse, continuing to provide smaller teacher-to-learner ratios and adequate feedback to learners. We describe below how, through longitudinal electives, M4 NPTs serve as information providers, learning facilitators, role models, and mentors.

### Anatomical Science Teaching Elective

The Anatomical Sciences Teaching Elective is offered to M4s interested in gaining experience teaching anatomy to pre-clerkship students. With an open registration (no application, no limit of accepted students), the course runs throughout M4 year. Students read and summarize articles on pedagogy and then complete 80 teaching hours (flexible scheduling throughout M4), selecting from a variety of teaching opportunities (i.e., facilitating in (neuro)anatomy laboratories, instructing ultrasound, clinical skills sessions, small and large group tutoring, assisting with anatomy practical examinations). All students complete a capstone, creating a tangible product in a variety of formats: narrated PowerPoints, practice quizzes for learner self-assessment, study guides, clinical skills video demonstrations, or presenting a lecture in M1/2 courses. M4 student-teachers are encouraged to select a topic that feels impactful for their future career and that they believe will enhance the UME experience for future students. Completed projects are peer-reviewed by other M4s within the elective, who provide a short, written evaluation.

As a strength, this teaching elective provides medical students an opportunity to serve as resource providers, facilitators, and mentors while learning about the art of teaching in a manner that is applicable to multiple disciplines. The challenges associated with the elective are minimal, given the flexibility awarded to the students (selecting/scheduling teaching sessions and capstone project).

### Clinical Teaching Elective

The clinical teaching elective (CTE) offered to M4s prepares students for their future roles as teachers in residency. Unlike the anatomy elective above, interested students must apply and be selected for this elective by the clinician educator elective director (based primarily on applicants’ interest in becoming clinician educators). The elective offers self-directed learning, with elective directors serving intentionally as mentors, rather than teachers. Each M4 student–teacher reads and reflects on educational modules (articles, websites, blogs, and/or podcasts; curated by elective directors) on MedEd pedagogy and adult learning theory. Students in the elective serve as peer instructors in the clinical skills (CS) course that they completed during their M1/2 years (eight teaching sessions required, 3–4 h each). Here, M4s observe M1s/M2s during simulated patient encounters and provides feedback through a post-encounter debrief. This allows the M4 to practice teaching principles learned in the modules, bolstering their own learning through practical application. In addition, the M4 student-teachers survey the student-learners weekly, resulting in 20–30 evaluations over the year. This feedback from near-peer learners is supplemented with direct narrative feedback from elective directors, who watch recorded videos of M4s teaching during the year.

One challenge encountered through this elective is students may be less equipped to teach certain material than faculty. While this can be overcome with adequate preparation, some M4s may lack awareness of what adequate preparation truly entails or may lack confidence in teaching even if prepared. Providing M4s with review material, objectives, and expectations may avoid this potential pitfall. In the CTE, objectives guide M4 preparation, and expectations are set during a pre-teaching brainstorm with elective directors.

Overall, year-long M4 teaching electives provide flexibility and resource support to students. These opportunities may create logistical challenges for elective directors, who must prepare for high variability in student–teacher aptitude and availability.

## Longitudinal Medical Education Experiences

Unlike electives contained within an academic calendar year, longitudinal experiences span several years (at UCCoM, from M1 to M4). With the benefit of time, these experiences provided increased opportunity for students to reflect and grow into their educator identity. Students are encouraged to explore the depth of MedEd — beyond practice and into scholarship. As such, most students who elect to apply for these opportunities voice a desire to work in academic medicine as a physician educator. Here, we describe two such offerings that encompass five of the six areas of “medical teacher” [8] while encouraging students to engage in scholarly projects in MedEd.

### Teaching in Medical Education (TiME): A Longitudinal Elective

The utilization of medical students as paid peer tutors is a well-documented strategy to bolster learning in UME [28, 29], with clear benefits for student-learners. However, few enumerate explicit training in *teaching* strategies. Over 90% of UCCoM M1 students and 60% of M2 students utilize peer tutoring. While the high demand demonstrates the value of this offering, one drawback noted in 2019 is that — since historically peer tutors were paid — the program was becoming financially unsustainable. As a means to alleviate the prohibitive cost while simultaneously addressing a need to formally introduce student-teachers to educational theory, a credit-based peer teaching longitudinal elective called “teaching in medical education” (TiME) was created.

Modeled after a “supplemental instruction” format [30], TiME was designed to assist M1 learning. More, however, TiME helps grow medical student-teachers’ knowledge about learning theory and pedagogy, improve students’ communication and professionalism, and apply teaching skills. Students apply as M1s (applications blinded and reviewed by students and faculty), and the 25 accepted students participate in teacher training through spring and summer. Training by the elective director (an educator faculty) and senior TiME students includes readings, podcasts, group discussions, and post-session reflections, through which students encounter content from education theory, to personal barriers in student learning, to teaching practice and teacher identity [8, 31–34].

Students begin teaching as M2s, running approximately 20 NPT sessions for M1 learners in the foundational and organ system courses. This completes most of the required 80 preparation/teaching hours for this longitudinal elective. Teaching requirement in the M3/M4 years is intentionally less (approximately 10 h each), to accommodate clerkship schedules. M3/M4 student-teachers run NPT sessions or engage in MedEd in other ways. For example, students can create projects that improve the TiME elective itself (i.e., teach pedagogy and education practice to M1s in the elective) or the UCCoM curriculum overall (i.e., create practice questions, study materials, supplemental learning opportunities, pedagogical videos).

M2–M4 student-teachers receive anonymous evaluations (aggregates from all teaching session) twice per academic year, which they review with the TiME elective director in a one-on-one feedback session during their M2 year. This allows the student–teacher to identify successes, discuss areas for improvement, and set goals for future teaching sessions.

Overall, the elective provides consistent and continuous opportunities for teaching (with feedback) while reducing the demand for paid tutoring. One concern was the burden student-teachers might experience while also being a student-*learner*. Indeed, students acknowledge that creating effective teaching sessions takes more time than anticipated; however, no students expressed this challenge hindered their own learning. In fact, many voiced an appreciation for the proximity of their student-teaching to the upcoming USMLE exam, implying that teaching helped bolster their own learning.

### Curricular Scholar Program for Medical Education

Scholarship concentration programs for medical students have been around for decades [35, 36]. These programs allow medical students to develop specialized expertise in an area of scholarship, critical thinking, and methods of investigation. Developing habits of self-directed lifelong learning and providing formal mentorship [37], they encourage curiosity in a particular area of medicine while concomitantly providing distinction for a student’s residency application portfolio [38, 39]. At UCCoM, we offer a medical education-focused medical student scholarship program (MedEd-MSSP).

The MedEd-MSSP provides highly motivated students with a unique opportunity to understand and experience specific interests within the MedEd continuum, throughout their 4 years of medical school. Interested students apply for the MSSP in their first semester of medical school (reviewed by the MSSP director). Two selected students begin the program in January (M1) and complete the program (250 h) by graduation (with annual checkpoints), conferring a medical degree with distinction in MedEd. MSSP students must dual enroll in one of the aforementioned MedEd-related electives (TiME or CTE). Program hours are fulfilled by conducting a faculty-guided scholarship project along with MedEd-focused didactics, journal clubs, participating in related service, and conducting formal instruction as an NPT.

MedEd is a broad discipline, and the MedEd-MSSP intends to expose students to that breadth; as such, students fulfill specific requirements in four domains of medical education: (1) assessment, (2) evaluation, (3) curriculum design/didactic teaching, and (4) clinical teaching. In other words, these scholar students will not be able to solely concentrate on their favorite domain to fulfill the requirements of the program but rather will have the opportunity to serve in several roles of the medical teacher [8].

Compared to stand-alone electives or extra-curricular offerings, the MedEd-MSSP provides a pathway for medical students who are willing to adopt medical education as a significant component of their career path. Temporally, starting the MSSP as an M1 provides a wealth of opportunities to assist in the identification of one’s passions. However, this early adoption also presents a challenge, as students who take an interest in MedEd in M2–M4 years are unable to enroll. Another noteworthy benefit is the individual attention to students, who work one-on-one with a faculty advisor for their scholarship project, creating a graduate school-like mentorship. Students receive a small stipend for conducting scholarly work in the summer after their M1 year, which may limit the number of accepted applicants. However, despite the challenge this may present (competition), the funded research model helps to maintain the robust nature of the program.

## Extra-Curricular Teaching Opportunities

Extra-curricular teaching allows students to share insights, observations, and lessons beyond “knowledge for practice” and can be tailored to a unique student experience. Peer education within a structured curriculum challenges student-teachers to master focused content and create resources, but extra-curricular teaching allows students to engage in the entire process of curriculum development with a long-term lens: developing content de novo, communicating and refining ideas, collaborating with faculty advisors, devising implementation strategies, and considering program evaluation. The initiatives described here are also student-led (faculty providing only essential guidance). Such autonomy allows students to engage fully with their peer learners, maximize the impact of content delivery, and modify their communication to interface with learners across the stages of student education.

### A Student-Generated Implicit Gender Bias Peer-to-Peer Curriculum

One extra-curricular initiative at UCCoM is a peer-to-peer (P2P) thread designed to mitigate implicit gender bias within the healthcare (education) system. Noting occurrences of gender bias within UCCoM’s UME curriculum, two medical students (guided by faculty) proposed to education administration a mandatory extra-curricular thread on gender bias be introduced to pre-clerkship students; the broad goals of this thread were to help students identify and discuss ways to effectively respond to implicit bias. The crux of this curriculum is brief (15 min) but high-impact, sessions (eight total sessions during pre-clerkship years). The nature of P2P session content ranges from real case studies experienced by students and faculty within our own medical school, to short-story vignette readings, to bystander training that prepares students to be allies during clinical years (when incidents of implicit bias increase at UCCoM). Ensuring these sessions are led by peers dissipates power differentials and allows for effective teaching of content not explicitly taught within the classroom [40]. Of note, administrative support was essential to label these extra-curricular sessions as “mandatory attendance” outside of the UCCoM-sanctioned foundational and clinical content.

One benefit of developing this curriculum from conception to practice was that the students learned how to formulate individual learning sessions that served a larger curriculum scheme, which involved managing logistics and working closely with educational leadership. To ensure sustainability of this student-led initiative and to continue to meet the evolving needs of subsequent student cohorts, the student co-creators developed a recruitment process. This represents one challenge with this teaching opportunity — the need to continually identify students that share the P2P founders’ passions. Being student-led, this requires a dedicated student leadership team, and — as with most students-as-teachers programs — the program may stall if students become overwhelmed with their own learning responsibilities.

### Medical Student Grand Rounds

Another student-generated endeavor is our Medical Student Grand Rounds (MSGR). Recognizing an abundance of passion-projects among UCCoM medical students, an alumnus suggested there should be opportunities for students to formally share their interests with peers. Thus, MSGR was founded and implemented with a primary goal: provide students an opportunity to create and deliver a grand rounds-style presentation on any topic they choose — however directly or tangentially it aligned with the formal medical school curriculum. Any student may apply to give an MSGR presentation, and talks are selected by a student committee, with preference given to topics that are perceived to be appealing to a large audience (i.e., how to appropriately use social media, effects of pet ownership on health, LGBTQ healthcare, caring for adult cancer survivors, the future of pain control, compassionate care, and unhealthy approaches to obesity).

Student experiences prior to medical school vary greatly, and MSGR provides an outlet for sharing from one’s unique expertise. For instance, the student who presented on obesity was a nutritionist prior to medical school. The student presenting on adult cancer survivors is a cancer survivor, themself. Other students built upon their interests by taking a deep-dive into the literature to educate others about that topic. Educator faculty advise each student to advance their skills in designing and delivering large group presentations. These interactions are intentionally minimal, as faculty intend only to provide guidance feedback — giving students primary control in crafting their seminar-style talk.

Both the P2P and MSGR programs were created *by* students *for* students and encourage students to share their unique interests in constructive and meaningful ways. Here, student participants primarily serve as information providers and resource creators; however, the programs also allow for planning and practice in curriculum development. Both have faculty support and guidance, which creates a need for faculty willingness to serve in this capacity. While the P2P curricular thread is a longitudinal undertaking and MSGR is focused on developing a single impactful session, both share similar challenges. First, students may need to collaborate with education leadership and learn to navigate administrative processes when providing content outside of the college-approved curriculum. For the mandatory P2P thread, multiple presentations were conducted with various UCCoM stakeholders prior to earning program approval. While students understand the value of administrative policy, this process may dissuade students from taking initiative. In addition, both MSGR and the P2P curricular thread challenged student-teachers to create content on sensitive topics that may be received differently by each medical student; thus, student-teachers must learn to convey information, address questions, and facilitate discussion mindfully in a manner that allows the audience to feel heard. Additionally, as mentioned above, the continued success of such programs requires intrinsic motivation and dedication of student-teachers, who must assemble meaningful presentations while balancing their own schedules. Committing to these initiatives can be difficult and has resulted in some sessions being postponed or fewer sessions being implemented overall. Finally, MSGR presentations are not mandatory, sometimes resulting in lower attendance, potentially detracting from the student–teacher’s overall experience.

## Summary

Teaching is an unavoidable aspect of all medical doctors — from residency to attending physicians. Lest we forget, doctor is derived from the Latin word, *docere*, meaning “to teach.” The ever-increasing number of publications regarding the importance of high-quality teaching on learning and retention necessitates opportunities for medical *students* to practice being *teachers*. Harden and Crosby describe the complex responsibilities of a medical teacher [8]. We describe a variety of options for medical students to assume these various roles, through diverse offerings at UCCoM with varied time commitment, faculty involvement, learner composition, and content focus. In totality, our teaching opportunities encompass five of the six areas (excluding “assessor”), with information provider being the most commonly practiced role [8] (Fig. 2). 

Perhaps the primary challenge one might anticipate when medical students become teachers is time. Indeed, dedication is required for any opportunity to be implemented effectively. Among learners, one’s ability to assume responsibilities is student-dependent, in a manner that is reflective of actual teaching practice. This necessary discernment is one experienced by real-world educators considering whether or not to assume additional teaching responsibilities. However, student-teachers at UCCoM do not find this balance to be unmanageable. They understand a need to reflect on their own bandwidth, and many who participate as teachers share a learned importance and practice of time management — a critical skill to gain for any health professional, across specialties. Some found the work associated with teaching was beneficial for the student-teachers, as they recognized a direct benefit in their preparation for “what’s next” in their MedEd journey (i.e., M2s found teaching beneficial for board exam preparation, and M4s felt prepared for their imminent resident teaching role). Even still, it is critical in the call for any students-as-teachers program that leaders clearly present the anticipated time commitment and speak realistically about the amount of effort students are expected to devote.

Overall, we encourage all medical schools to consider developing one or more of these valuable MedEd programs. This can be accomplished through a variety of opportunities offered at different time points in a medical school curriculum (Fig. 1), which can easily be adopted by and adapted for other health professions schools. It is possible to meet the needs of students who express varying desires to become a medical teacher, from those with known career aspirations as clinician faculty educators, to those who are just beginning to exploring MedEd, to those who have no desire to become a formal educator, yet want to ensure they are a competent resident-teacher. Notably, the faculty serving as advisors or teacher trainers in each of UCCoM’s programs are *educator* faculty and thus can guide students in incorporating teaching experience(s) into one’s professional identity.

Finally, the described courses demonstrate a spectrum with regard to resources required to run the course, which can be altered to suit the needs of another institution. We note that certain challenges exist in all courses (i.e., content expertise), while other challenges are unique to the specific course offering (i.e., scheduling conflicts). Nonetheless, the variety of experiences allows any interested student a pathway toward development as a medical educator, directly addressing the need for earlier exposure to and experience with teaching practice.
